# MALDI MSI Protocol for Spatial Bottom-Up Proteomics at Single-Cell Resolution

**DOI:** 10.1021/acs.jproteome.4c00528

**Published:** 2024-10-24

**Authors:** Andrej Grgic, Eva Cuypers, Ludwig J. Dubois, Shane R. Ellis, Ron M. A. Heeren

**Affiliations:** † The Maastricht MultiModal Molecular Imaging (M4I) Institute, Division of Imaging Mass Spectrometry (IMS), Maastricht University, 6229 ER Maastricht, The Netherlands; ‡ The M-Lab, Department of Precision Medicine, GROW − Research Institute for Oncology and Reproduction, Maastricht University, 6229 ER Maastricht, The Netherlands; § Molecular Horizons and School of Chemistry and Molecular Bioscience, University of Wollongong, Wollongong, New South Wales 2522, Australia

**Keywords:** MALDI, MSI, mass spectrometry imaging, high spatial resolution, single-cell, proteomics

## Abstract

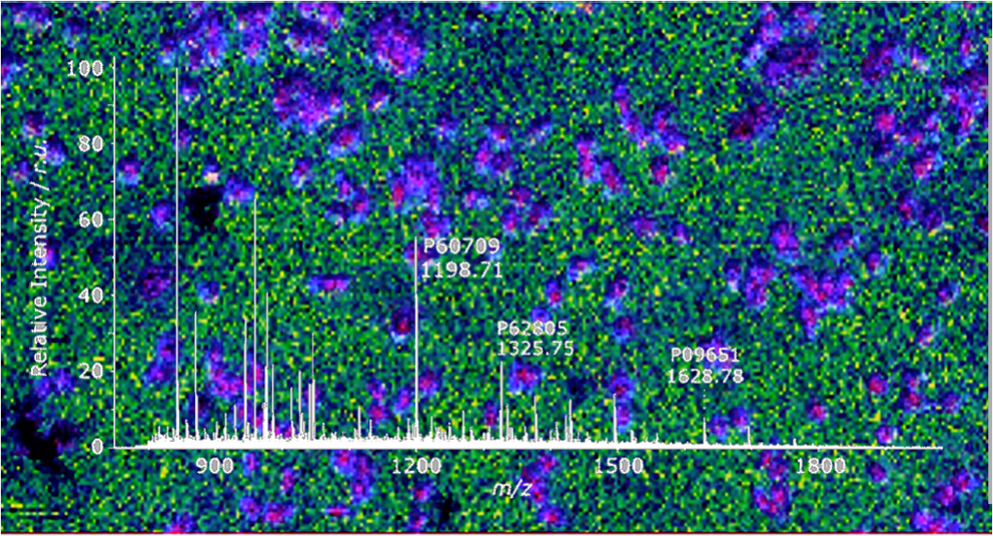

Matrix-assisted laser desorption/ionization (MALDI) mass spectrometry imaging (MSI) started with spatial mapping of peptides and proteins. Since then, numerous bottom-up protocols have been developed. However, achievable spatial resolution and sample preparation with many wet steps hindered the development of single cell-level workflows for bottom-up spatial proteomics. This study presents a protocol optimized for MALDI-MSI measurements of single cells within the context of their 2D culture. Sublimation of CHCA, followed by a dip in ice-cold ammonium phosphate monobasic (AmP), produced peptide-rich mass spectra while maintaining matrix crystal sizes around 400 nm. This enables MALDI-MSI imaging of proteins in single cells grown on an ITO slide with a throughput of approximately 7800 cells per day. 89 peptide-like features corresponding to a single MDA-MB-231 breast cancer cell were detected. Furthermore, by combining the MALDI-MSI data with LC-MS/MS data obtained on cell pellets, we have successfully identified 24 peptides corresponding to 17 proteins, including actin, vimentin, and transgelin-2.

## Introduction

As technology advances, the focus of the mass spectrometry imaging (MSI) community has been shifting from analyzing tissues in bulk to analyzing on a single-cell level.^1^ The applicability of matrix-assisted laser desorption/ionization (MALDI)-MSI has been demonstrated for single-cell analysis of lipids, small metabolites, and intact proteins.^2−5^ Visualizing protein distributions on the single-cell scale has been mainly done with labeling techniques such as metal-based immunohistochemistry (IHC) and imaging mass cytometry.^6^ MALDI-IHC has recently been used for high spatial resolution proteomics measurement in combination with MALDI-MSI measurement of N-Glycans.^7^ However, there is still a lack of untargeted and nonlabeled proteomic imaging approaches that offer good sensitivity at the single-cell level.

LC-MS/MS-based single-cell proteomics (SCP) is a rapidly growing research field.^8^ Currently, the most sensitive methods performed with either Orbitrap or timsTOF instruments can identify and quantify around 2000 proteins per single cell with a throughput of 40 single cells per day for timsTOF and 80 for Orbitrap.^9,10^ Other techniques, such as plexDIA, offer much higher throughput at approximately 144 single cells a day while still quantifying around 1000 proteins per cell.^11^ MALDI-based SCP approaches offer significantly higher throughput and have demonstrated the ability to analyze up to 3000 cells per hour.^12^ Even mass spectra corresponding to a single organelle can be obtained with this technique.^13^ However, as single cells and organelles are isolated and placed on an ITO-coated slide, there is, as well as with LC-MS/MS-based SCP, a loss of spatial context of cells within their native environment and distributions of proteins within the cells.

MALDI-MSI has the potential to offer high throughput while imaging single cells in the context of their 2D cell culture or tissue environment, offering a direct correlation between cell morphology and mass spectra. Development of novel optical designs has resulted in MALDI ion sources allowing sub 5 μm laser spot size.^2−4^ This has led to the imaging of native proteins at a spatial resolution of 1 μm.^5^ However, the development of spatial bottom-up proteomics methods was hindered by the high number of wet steps in the protocol, which caused delocalization, and the lack of suitable matrix sublimation protocols.

All published on-tissue digestion workflows generally have at least four wet steps: protein fixation, washing out lipids and small metabolites, trypsin application, and matrix application.^14−17^ Protocols can include additional wet steps, such as antigen retrieval or recrystallization, to enhance the analyte signal. However, these wet steps combined make it difficult to perform bottom-up proteomics on a single-cell level, as endogenous analytes tend to diffuse in a wet environment.

While other protocols focused on optimizing washing steps, enzyme application, and matrix spraying methods, none focused on developing a solvent-free matrix application method. Solvent-free matrix application results in smaller crystal sizes, making sublimation an excellent option for high spatial resolution measurements.^18^ However, sublimation has been shown to decrease the number of observed analytes.^18^

This paper presents an optimized protocol for high spatial resolution MALDI-MSI bottom-up proteomics. Wet steps were optimized to reduce analyte delocalization during the on-tissue digestion process. A CHCA matrix sublimation method was developed, resulting in small matrix crystals and reduced delocalization. After matrix sublimation, an ammonium phosphate monobasic (AmP) dip was performed to achieve a high-quality peptide mass spectrum. Finally, the optimized protocol was utilized for bottom-up proteomics MALDI-MSI measurements of fresh-frozen mouse kidney section and single cells (MDA-MB-321 breast cancer cell line). LC-MS/MS measurements of digested cell pellets were performed to identify detected peptide-like features from MALDI-MSI mass spectra.

## Materials and Methods

### Chemicals

Water (HPLC/MS grade), ethanol, acetonitrile (ACN), formic acid (FA) and acetone were obtained from Biosolve BV (Valkenswaard, The Netherlands). α-Cyano-4-hydroxycinnamic acid (CHCA), ammonium phosphate monobasic (AmP), ammonium formate, ammonium bicarbonate (ABC), dithiothreitol (DTT), iodoacetamide (IAM), poly-l-lysine, and potassium nitrate were obtained from Sigma-Aldrich (St. Louis, MI). Sequencing grade Modified Trypsin and Trypsin/Lys-C mixture were acquired from Promega (Madison, WI).

### Samples

A fresh-frozen mouse kidney (*n* = 1) was obtained from the Johns Hopkins University School of Medicine. The kidney was sectioned at 12 μm thickness and mounted on indium tin oxide (ITO) coated glass slides (Delta Technologies Ltd., Loveland, CO). All animal experiments were performed with appropriate ethical approval (A3272-01 at Johns Hopkins University) and in compliance with the respective institutional guidelines. The cell line MDA-MB-321 was purchased from Leibniz Institute DSMZ (Braunschweig, Germany).

### Cell Preparation on Slides

Slides were prepared based on the protocol by Cuypers et al.^19^ Briefly, cells were grown in 90% Dulbecco’s Modified Eagle Medium (DMEM) and 10% fetal bovine serum (FBS) medium. ITO glass slides were coated with 20 μL of poly-l-lysine (1:1 dilution with water). Slides were washed with water and placed in a Petri dish (conductive side facing up). Approximately 10^6^ cells were added to the Petri dish and incubated overnight at 37 °C with 5% CO_2_. Two phosphate-buffered saline washes followed the removal of the growth medium. Neutral-buffered formalin (10%) was added for 10 min. Slides were washed twice with 50 mM ammonium formate and twice with MQ water. Finally, the slides were dried under a gentle nitrogen gas stream.

### Optical Microscopy

Optical images were obtained at 20× magnification using a Leica DMRX optical microscope (Leica Biosystems Imaging, Germany) equipped with a Nikon digital camera DXM1200 (Nikon, Japan).

### On-Tissue Trypsin Digestion

All slides were washed and fixated in ice-cold 100% ethanol (2 × 2 min), ice-cold 96% ethanol (1 × 1 min), ice-cold 70% ethanol (1 × 1 min), and 4 °C HPLC water (2 × 2 min). The slides were subsequently dried in a desiccator, and teaching points were applied with a white paint marker. Slides were scanned before antigen retrieval. Antigen retrieval was performed using the Retriever 2100 (Aptum Biologics, Rownhams, U.K.) for 20 min at 121 °C. The HPLC-grade water was used as a buffer for antigen retrieval. The slide holder was removed from the antigen retriever and cooled in an ice bath for 5 min. Half of the buffer was replaced with HPLC-grade water, and the slide holder was placed back to cool further in an ice bath. This was repeated twice, after which the slides were dried in a desiccator. The trypsin solution was prepared fresh by adding 200 μL of the cold HPLC-grade water to 20 μg of trypsin. The enzyme was sprayed with an HTX M3+ sprayer (HTX Technologies, Carrboro, NC). Spraying parameters were as follows: temperature = 45 °C, nozzle velocity = 1200 mm/min, flow rate = 30 μL/min, trypsin concentration = 0.1 μg/μL, number of passes = 8, track spacing = 2.5 mm, nitrogen gas pressure of 10 psi. The slide was then put in an incubation chamber at 37.5 °C for 16 h. The incubation chamber was prepared as described by Grgic et al., except that potassium nitrate was used instead of potassium sulfate to further minimize delocalization.^17^

### Matrix Application

For sublimation of CHCA, 50 mg of CHCA was dissolved in approximately 1.5 mL of acetone using a sonication bath. An HTX sublimator (HTX Technologies, Carrboro, NC) was used for the matrix sublimation. The CHCA solution was applied to the heated tray (60 °C) of the sublimator with a pipette to achieve homogeneous coverage. The matrix was sublimed at 170 °C for 10 min. After sublimation, slides were dipped in the ice-cold 100 mM AmP solution and dried vertically in a desiccator. No more than two slides were immersed in the same AmP solution to avoid cross-contamination.

### MALDI-MSI Measurements

All MALDI-MSI measurements (*n* = 3) were carried out with timsTOF flex (Bruker Daltonik GmbH, Bremen, Germany) in positive ion mode with a pixel size of 5 μm. The number of laser shots per pixel was 200, the laser frequency was 5 kHz, and the *m*/*z* range for all measurements was 800 to 2500. The figures display only segments of the *m*/*z* range that contain peptide-related spectral features.

### Data Analysis

Data analysis was performed with SCiLS lab 2022a (SCiLS GmbH, Bremen, Germany). Generated images were all normalized to the root-mean-square (RMS) and exported from the SCiLS lab. Acquired mass spectra were exported from the SCiLS lab and processed with mMass software.^20^ MALDI-TOF Peptides settings were used for baseline correction in the mMass software. This was followed by peak picking utilizing the following settings: relative intensity threshold of 4%, picking height of 75%, and applied deisotoping. Any leftover isotopic peaks were removed manually. Data was recalibrated using trypsin autolysis peaks in every scan (*m*/*z* 842.51, *m*/*z* 870.54, and *m*/*z* 1045.56).

To tentatively identify spectral features as “peptide-like,” the generated peak list was evaluated based on two conditions. First, the mass defect of the peak must align with trends observed for tryptic peptides.^21^ Second, the peptide must be observed in multiple cells. Peaks that did not meet the criteria were excluded to prevent artificially inflating the number of actual detected peptide features, resulting in reported 89 peptide-like features.

The throughput (*T*) of this method was calculated using the following equation:
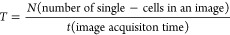


### Scanning Electron Microscopy (SEM) Imaging

SEM imaging was used to determine the CHCA matrix crystal size with and without the AmP dip. After the CHCA matrix sublimation, half of the slide was dipped in an ice-cold 100 mM AmP solution. Then, an ITO slide was mounted on a stub and placed into the JEOL JSM-IT200 Scanning Electron Microscope. No additional preparation was needed as the slides are conductive due to the ITO coating.

### Peptide Identification with LC-MS/MS Bottom-Up Proteomics

Liquid sample preparation was performed as described by Gundry et al.^22^ Briefly, a buffer containing 5 M urea and 50 mM ABC was added to the cell pellet. Cell lysis was performed by three freeze–thaw cycles, using dry ice for freezing and sonication (40 s) in an ultrasonic bath for thawing. Protein concentrations were assessed using the Bradford assay, and equal amounts of protein were used for analysis (50 μg per sample). Protein lysates were then reduced with 20 mM DTT for 45 min, followed by alkylation with 40 mM IAM for 45 min in the dark. The alkylation was terminated by adding 20 mM DDT to consume excess IAM. In-solution digestion was performed with a Trypsin/LysC mixture, added at a 1:25 (enzyme: protein) ratio, for 2 h at 37 °C in a Thermo Shaker (Grant Instruments, Shepreth, UK) at 300 rpm. Next, the lysate was diluted to 1 M Urea using 50 mM ABC and further digested at 37 °C at 300 rpm overnight. The addition of FA to a total of 1% terminated the digestion.

Peptide separation was performed on a Thermo Scientific (Dionex) Ultimate 3000 Rapid Separation ultrahigh-performance liquid chromatography (UHPLC) system equipped with a PepSep C18 analytical column (15 cm, ID 75 μm, 1.9 μm Reprosil, 120 Å). Before UHPLC separation, tryptic peptides were desalted on an installed C18 trapping precolumn. Peptides were separated on the analytical column using a 90 min linear gradient of acetonitrile (5–35%) with 0.1% FA at a 300 nL/min flow rate. Mass spectra were collected on a Q-Exactive HF mass spectrometer (Thermo Fisher Scientific, Bremen, Germany) using a data-dependent acquisition method. Raw mass spectra were processed in Proteome Discoverer software (version 3.0, Thermo Fisher Scientific, Bremen, Germany). Protein identification was performed using the SEQUEST search engine and the SwissProt Human database (SwissProt TaxID = 9606, v2017-10-25).

After recalibration, the *m*/*z* values obtained with MALDI-MSI were matched with the theoretical *m*/*z* values for the [M + H]+ form of the peptides detected with LC-MS/MS. Only protonated adducts were considered in the MALDI MSI data set to minimize false positives, and no more than three missed cleavages per peptide were allowed. Finally, a match was confirmed if the MALDI-MSI *m*/*z* value was ±5 ppm of the *m*/*z* values obtained with the LC-MS/MS.

## Results and Discussion

### Optimization of the Trypsin On-Tissue Digestion Protocol for High-Spatial Resolution Measurements

As described in previous publications, ice-cold solvents minimize the amount of delocalization during the wash and fixation steps.^14,17^ Proteins are then denatured in the antigen retrieval steps to expose more cleavage sites, leading to more efficient on-tissue digestion, by heating samples to 121 °C.^17^ Slides were then dried under vacuum before enzyme application with the HTX M3+ sprayer to prevent additional delocalization caused by nitrogen gas flow. Enzymes were applied with the HTX M3+ sprayer to achieve a uniform trypsin application (Figure S1, Supporting Information). A saturated potassium nitrate solution kept the relative humidity lower and more stable during overnight incubation.^23^ Incubation time was ∼16 h to achieve maximum cleavages while keeping experimentation time within reason.

### Optimization of the CHCA Matrix Sublimation Method

Based on in-house optimization of CHCA spraying parameters, the best achievable spatial resolution was around 10 μm. While 10 μm can already be considered a high spatial resolution, it is a factor of 4 larger than measurements at 5 μm, which can be performed with commercially available instruments.^2−4,24,25^ As matrix spraying is a solvent-based matrix application method, it introduces analyte delocalization, which prevents measurements at 5 μm even when the matrix crystal size is significantly smaller than the pixel size (Supporting Information, Figure S2).^26^

**Figure 1:**
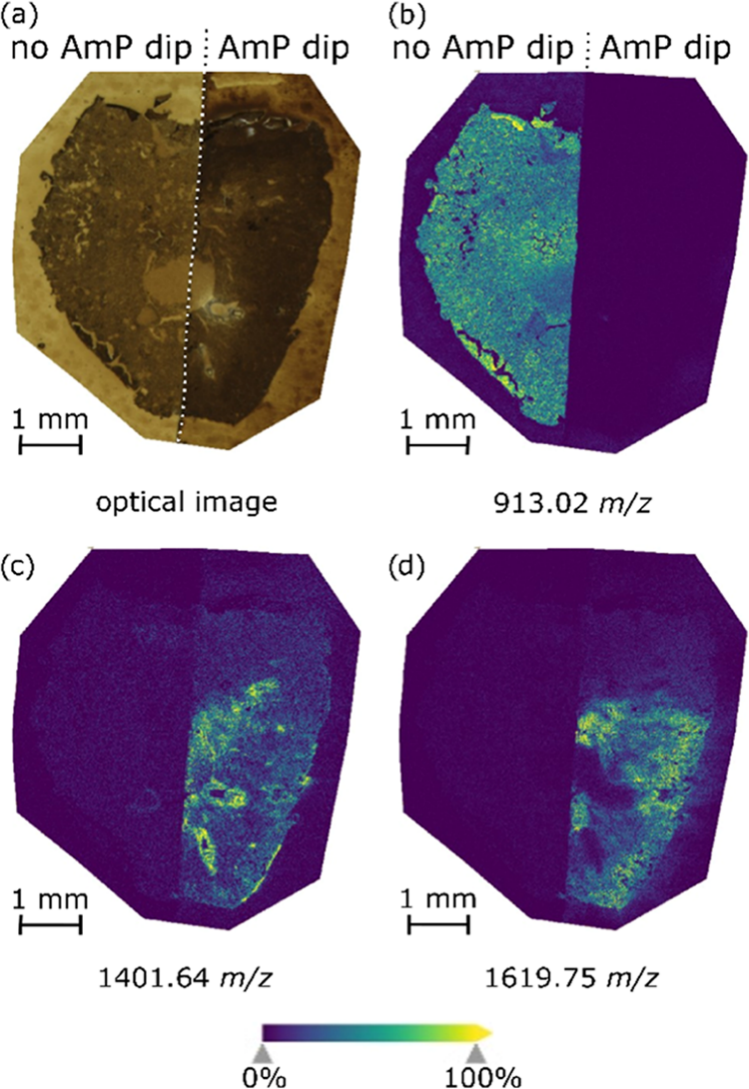
(a) Optical image of the kidney after dipping half of the kidney section in the AmP dip. (b) MALDI-MSI image shows matrix cluster-related peak spatial distribution almost exclusively on the nondipped side of the kidney section. (c) and (d) MALDI-MSI images show various spatial distributions of peptide peaks that correlate well with observed kidney section morphological features.

Once CHCA is dissolved in a minimal amount of acetone, it is transferred to a preheated tray of the HTX sublimator with a pipette. This leads to a homogeneous matrix deposition on a tray, which is crucial for homogeneous coverage of the slide after sublimation. When transferring matrix solution in one step, the solution evaporates faster on places on the heated tray just above the heating element; with a pipette, droplets can be applied at desired spots until the whole tray is homogeneously covered. After 10 min of sublimation at 170 °C, slides are covered with a yellowish, almost transparent matrix layer. AmP dip is then performed to suppress CHCA clustering and enhance peptide signal.^27,28^ An ice-cooled AmP solution is used for a maximum of two slides to minimize delocalization and cross-contamination. As demonstrated in Figure 1, the AmP dip is a crucial step that ensures this matrix sublimation protocol performs well. The selection of fresh-frozen kidney sections as the test sample was based on the anticipated robust tryptic peptide mass spectra following on-tissue digestion. Additionally, many morphological features inherent to kidney tissue could be used to assess potential delocalization.

**Figure 2:**
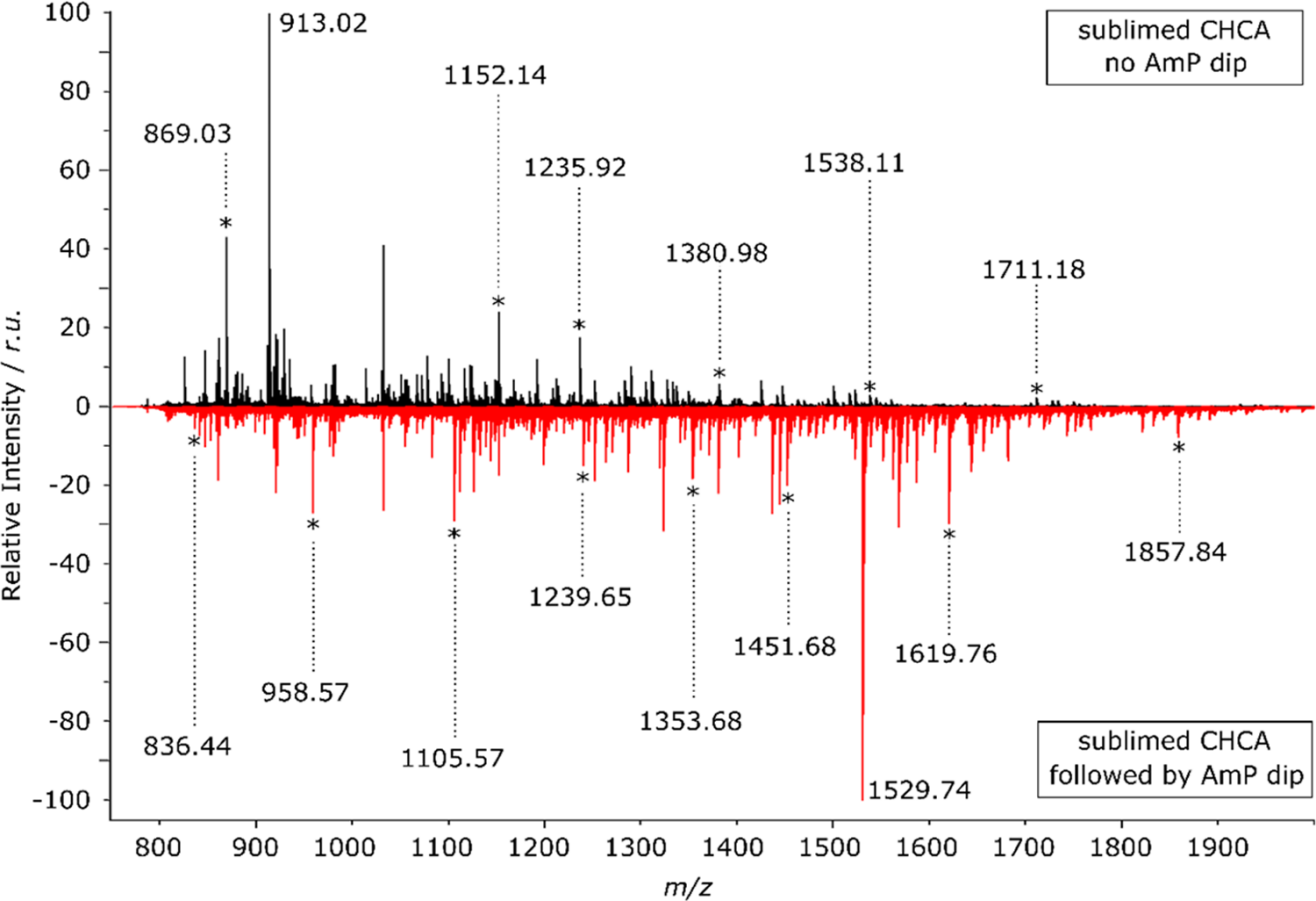
Averaged mass spectra representative of the nonimmersed (black) and immersed (red) regions of the kidney section, respectively. The introduction of the AmP dip generates peptide-abundant mass spectra, while the absence of the AmP dip results in a CHCA matrix cluster peaks dominated mass spectra.

Half of the fresh-frozen kidney tissue section was dipped in the AmP solution to enable a direct comparison using the other half of the tissue, which was not submerged in the AmP dip. A boundary between the undipped and dipped sections is visible on the optical image as the matrix layer turns from an almost transparent yellow layer to a thicker, darker matrix layer. As demonstrated in Figure 1, peaks with *m*/*z* values corresponding to matrix peaks show spatial distribution almost exclusively on the nonimmersed side of the kidney section. In contrast, observed peptide peaks are detected only on the AmP-immersed side of the kidney section. Furthermore, as shown in Figure 2, significant differences in mass spectra are observed after introducing the AmP dip. Matrix cluster peaks dominate the average mass spectrum corresponding to the undipped part, while almost no peaks could be tentatively assigned as peptides. On the other hand, virtually no matrix cluster peaks are observed in the averaged mass spectrum of the dipped part, as it produces a peptide-abundant mass spectrum.

**Figure 3:**
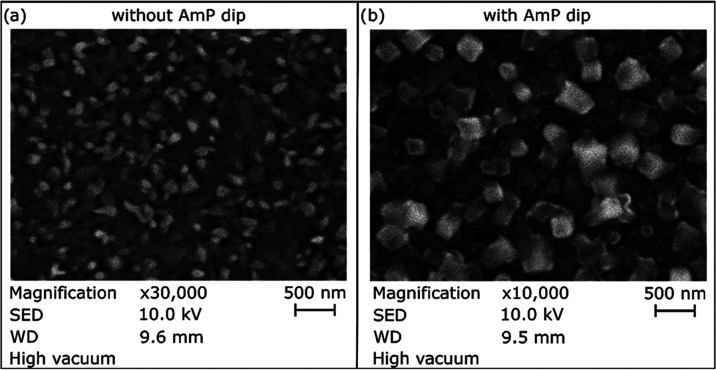
SEM images of matrix crystals were acquired with a JEOL JSM-IT200 Scanning Electron Microscope. (a) The SEM image of the CHCA matrix after sublimation with no AmP dip. (b) The SEM image of CHCA matrix crystals after sublimation was followed by an AmP dip.

As the matrix visibly changed color after the AmP dip, going from a yellowish, almost transparent layer into a thicker, almost white layer, the crystal shape and size were checked with SEM imaging. As shown in Figure 3, the crystals increased in size and got a more cubic shape. These changes are the most probable reason for the change visible to the naked eye and on optical images. Most importantly, even after the dip crystal size is well below 5 μm, more precisely, on average, it is ∼400 nm per crystal compared to the ∼100 nm without AmP dip (Supporting Information, Figure S3). While crystal size has increased almost 4 times after the AmP dip, matrix crystals produced with this method are more than suitable even for measurements at 1 μm with transmission-mode MALDI ion sources.^4^

### MALDI-MSI Imaging of Single-Cell Slides

Single-cell slides were treated with the optimized trypsin on-tissue digestion protocol followed by the developed CHCA sublimation method. The samples were imaged on a timsTOF flex in positive-ion mode to obtain an image of peptides released by on-tissue digestion. Three replicates of digestion on single-cell slides were performed, and MALDI-MSI measurements showed similar results.

**Figure 4:**
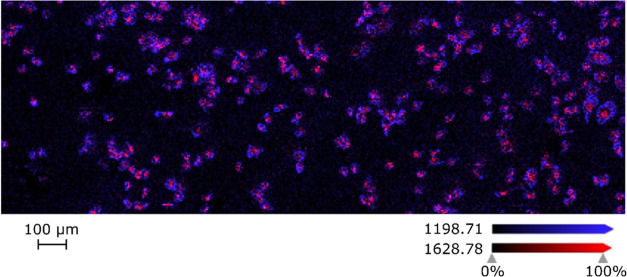
Overlay of two *m*/*z* values representing the two spatial distributions to which all detected peptides can be assigned. In this case, the spatial distribution of actin (*m*/*z* 1198.71, blue) and heterogeneous nuclear ribonucleoprotein A1 (*m*/*z* 1628.78, red) are shown. MALDI-MSI images were acquired on the timsTOF flex in positive mode at 5 μm spatial resolution.

Figure 4 demonstrates successful imaging of single cells after on-tissue digestion protocol at 5 μm pixel size. The obtained MALDI-MSI images can easily detect single cells in the context of the 2D cell culture. There is a good agreement in MDA-MB-231 cell size observed with MALDI-MSI and optical microscopy, varying in size from approximately 20 to 60 μm (Figure S4, Supporting Information). A throughput of 326 single cells per hour was achieved. If the average cell density across the entire ITO slide matches that of the measured area, approximately 7800 single cells could be measured per day.

Observed peaks in the area without cells correspond to trypsin autolysis peaks (Figure S1, Supporting Information). Trypsin autolysis peaks are useful because their identity is well-known and appear in almost every scan. Due to that, they can be used for data recalibration after measurements.^29^ Other interesting spatial distributions relate to the peptide distribution within the single cell. As shown in Figure 4, one of those distributions seems to correlate well with the cell membrane (blue color), and others are colocalized with the cytoplasm and nucleus inside the cell (red color). Speculations about the meaning of observed spatial distribution are supported by identifying the peak at *m*/*z* 1198.71 (blue) as a peptide specific for actin. Actin is known to be a significant cytoskeletal protein whose filaments are particularly abundant beneath the plasma membrane, enabling cells to migrate, engulf particles, and divide.^30^ Almost all other detected peptides seem to colocalize with the cell cytoplasm and nucleus, which is expected as protein concentration in the cytoplasm is estimated to be around 100 mg/mL.^31^ One of the proteins found inside the cell is heterogeneous nuclear ribonucleoprotein A1 (hnRNP A1). This protein, along with hnRNP A2, is the most abundant nuclear protein and moves between the nucleus and the cytoplasm.^32^ It is suggested that hnRNP A1 may regulate the expression of genes as it binds to double-stranded DNA.^33^ Additionally, it interacts with hormone response elements and other regulatory elements.^33^ The distinction between these two areas, presumably colocalized with the cell membrane and cytoplasm, can also be observed in distinct mass spectra on a single-pixel level (Figure S5, Supporting Information).

**Figure 5:**
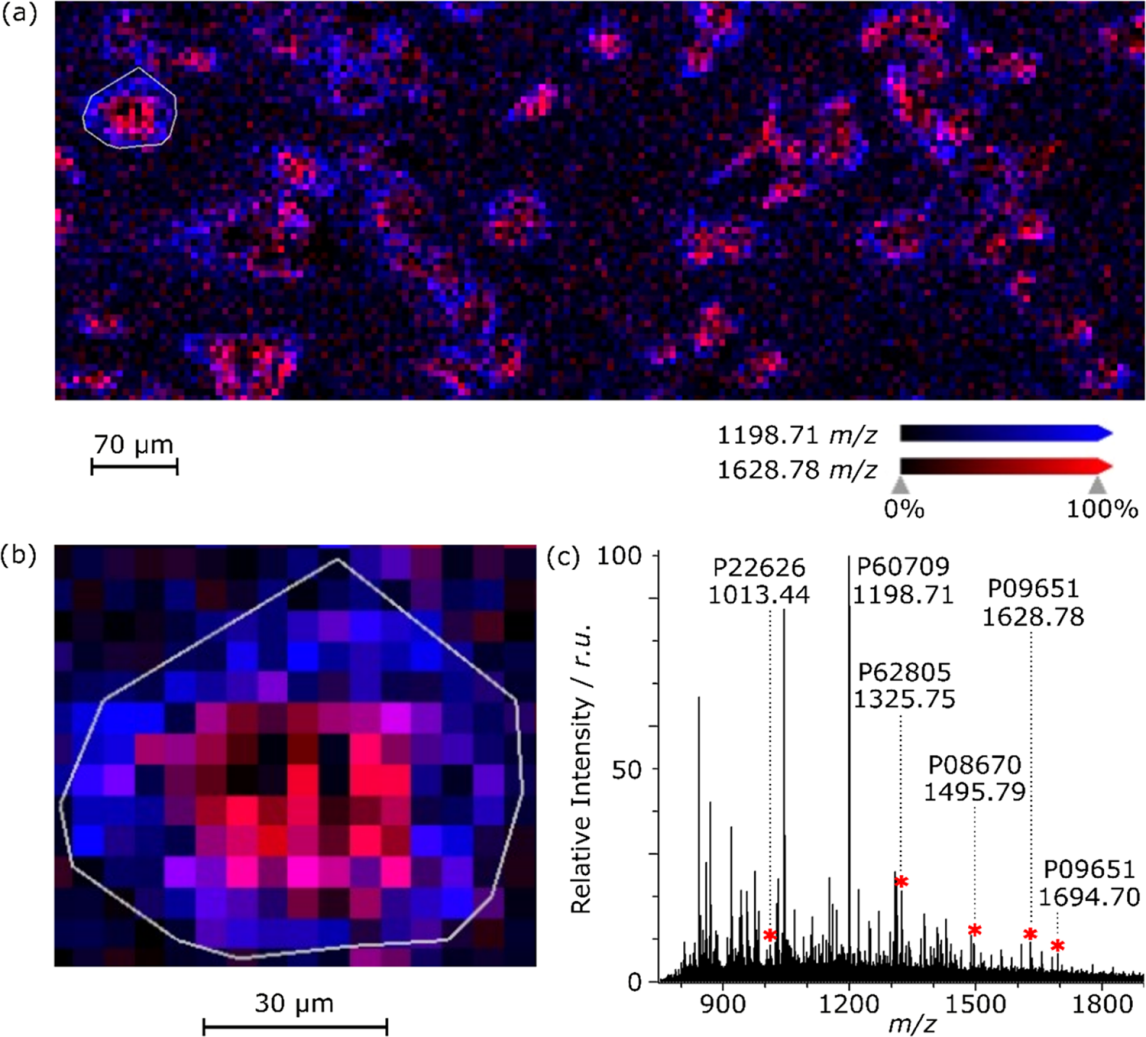
(a) Selected region of interest (ROI) is marked with white borders in the context of a single-cell slide it is on. (b) Zoom in on a selected ROI (single cell). (c) The average mass spectrum that corresponds to the selected ROI based on 137 averaged mass spectra. Additional single-cell images (optical and MALDI-MSI) are available in the Supporting Information (Figure S4).

Figure 5 shows an average mass spectrum corresponding to a representative single cell. This peptide-abundant mass spectrum results from averaging all 137 pixels of this specific cell. Based on their fractional mass, 89 observed peaks were tentatively identified as potential peptides.^21,34,35^ Out of 89 observed peaks in the spectrum, 24 peaks have been successfully identified with LC-MS/MS after trypsin digest of corresponding cell pellets. These 24 peaks correspond to 17 proteins, as some proteins have multiple corresponding peptide peaks detected. All detected peptides, peptide sequences, and corresponding proteins are shown in Table S1 (Supporting Information).

The main obstacle to the on-slide identification of peptides with MALDI-MSI is the low fragmentation yields of peptides for MS/MS due to their low charge states and the transient nature of the signal when scanning across heterogeneous surfaces.^36^ An additional problem is the bias of MALDI toward peptides with 1 or more missed cleavages, as extra lysine (K) and especially arginine (R) residues make peptides more ionizable.^37^ This is noticeable as no peptides with 0 missed cleavages are detected with MALDI-MSI (Table S1). From identified MALDI-MSI peptides, ∼63% had 1 missed cleavage, ∼33% had 2 missed cleavage, while 1% had 3 missed cleavage. Furthermore, every detected peptide had at least one arginine, while 50% of identified peptides had no lysine in their sequence. This indicates that with MALDI-MSI, we might miss all peptides with no missed cleavages or arginine in their sequence, as they may not be sufficiently ionizable. Lastly, an additional challenge when it comes to the identification of peptides observed with MALDI-MSI are isobaric species. However, coupling ion mobility has been shown to help resolve the isobaric species and increase the number of identified peptides on-tissue with MALDI-MSI experiments.^38^

## Conclusions

Here, we have presented a novel high-spatial resolution bottom-up proteomics protocol for MALDI-MSI. The sublimation of the CHCA matrix, followed by an ice-cold AmP dip, was a crucial step for successful high-resolution imaging experiments, yielding peptide-rich mass spectra even at a pixel size of 5 μm. Matrix crystals produced with this protocol have an average size of ∼400 nm, suitable for measurements with pixel sizes smaller than 5 μm, the current limit of commercially available instruments. The AmP dip helps with imaging biological samples with MALDI-MSI by preventing cluster formation of CHCA, lowering matrix ion suppression effects, and enhancing peptide signal intensity. Due to these improvements, it was possible to image single cells grown on an ITO slide. Those cells displayed two biologically relevant spatial distributions; one colocalized more with the cell membrane and the other with cytoplasm and nucleus inside the cell, where a higher abundance and diversity of proteins can also be noticed. MALDI-MSI can potentially analyze up to 7800 cells per day, offering a 54-fold increase in throughput compared to LC-MS/MS-based SCP, though it provides significantly lower qualitative and quantitative coverage of the cellular proteome. It does, however, provide spatial context for proteins within the cell and their native environment, which is not offered by LC-MS/MS or other MALDI-based approaches. Out of 89 peptide peaks observed in an averaged MALDI-MSI mass spectrum of one whole cell, 24 have been identified using LC-MS/MS, which confirmed the identities of 17 proteins. This protocol can potentially be a valuable tool for investigating changes in the proteome and identifying potential therapeutic targets within tissues, considering the heterogeneity of the cell population. It could also be helpful in the MALDI-MSI-based digital pathology field for cell classification and for studying cell–cell interactions in 2D cell cultures.

## Supporting Information Available

The mass spectrometry proteomics data have been deposited to the ProteomeXchange Consortium via the PRIDE partner repository with the data set identifier PXD056528. Table S1. List of peptides identified after combining MALDI-MSI data with LC-MS/MS data from cell pellets. Figure S1. The MALDI-MSI image of trypsin autolysis peak at *m*/*z* 842.51. Figure S2. SEM measurements of matrix crystals after spraying of CHCA matrix and corresponding MALDI MSI measurement of single cells. Figure S3. SEM measurements of matrix crystals after sublimation of CHCA matrix with and without AmP dip. Figure S4. Optical microscopy images and MALDI mass spectrometry images of MDA-MB-231 single cells on ITO-coated slides. Figure S5. Examples of single scans obtained from single cells at a pixel size of 5 μm (PDF)

## Abbreviations

MALDI: matrix-assisted laser desorption ionization
MSI: mass spectrometry imaging
LC-MSMS: liquid chromatography-tandem mass spectrometry
IHC: immunohistochemistry
ITO: indium tin oxide
SEM: scanning electron microscopy
CHCA: α-Cyano-4-hydroxycinnamic acid
ACN: acetonitrile
FA: formic acid
AmP: ammonium phosphate monobasic
ABC: ammonium bicarbonate
DTT: dithiothreitol
IAM: iodoacetamide
DMEM: Dulbecco’s modified Eagle medium
FBS: fetal bovine serum medium
