# Supplementary material for: MALDI MSI Protocol for Spatial Bottom-Up Proteomics at Single-Cell Resolution
Source: J Proteome Res. 2024 Oct 24;23(12):5372–9. doi: 10.1021/acs.jproteome.4c00528 (PMC11629377; doi:10.1021/acs.jproteome.4c00528)
Supplement: Supplementary file 1 — pr4c00528_si_001.pdf [file pr4c00528_si_001.pdf]

Supporting Information

MALDI MSI method for bottom-up proteomics at single-cell spatial resolution

Andrej Grgic[1], Eva Cuypers[1], Ludwig J. Dubois,[2] Shane R. Ellis[1,3], Ron M. A. Heeren[1]*

1 The Maastricht MultiModal Molecular Imaging (M4I) Institute, Division of Imaging Mass Spectrometry (IMS), Maastricht University, 6229 ER, Maastricht, The Netherlands

2 The M-Lab, Department of Precision Medicine, GROW – Research Institute for Oncology, Maastricht University, 6229 ER, Maastricht, The Netherlands

3 Molecular Horizons and School of Chemistry and Molecular Bioscience, University of Wollongong, Wollongong, New South Wales 2522, Australia

*To whom correspondence should be addressed

r.heeren@maastrichtuniversity.nl

Keywords: MALDI, MSI, mass spectrometry imaging, high spatial resolution, single-cell, proteomics

Table of Contents

Table S1. List of MALDI MSI peptide-like features identified via LC-MS/MS with corresponding peptide sequence, protein ID, and protein name. ……………….……….………1

Figure S1. Spatial mapping of the trypsin autolysis peak at *m/z* 842.51. Uniform slide coverage of trypsin was achieved with the HTX M3+ sprayer. The regions devoid of signal on the MALDI image correspond to individual cells cultivated on an ITO slide, where the autolysis rate is comparatively slower due to the presence of other proteins. The timsTOF flex instrument was used to acquire this MALDI MSI image at a spatial resolution of 5 µm in positive mode. ……………………………………………………………………………...…….…………2

Figure S2. (a) Images of the CHCA crystals after spraying with and without the AmP dip. (b) MALDI mass spectrometry images of single MDA-MB-231 breast cancer cells acquired with the timsTOF at 5 µm spatial resolution in positive ion mode and 250 laser shots per pixel. (c) The average mass spectrum that corresponds to the selected ROI based on 240 averaged mass spectra. ………………………………………………………………………………...……3

Figure S3. (a) Images of the CHCA crystals after sublimation and no AmP dip. The image corresponds to Figure 2a, presenting measurements of matrix crystals. (b) Images of the CHCA matrix crystals after sublimation and AmP dip. The yellow dashed rectangle corresponds to Figure 2b, presenting measurements of matrix crystals………………………………………4

Figure S4. Optical microscopy images of various single MDA-MB-231 cells on ITO-coated slides. (b) MALDI mass spectrometry images of various single MDA-MB-231 cells acquired with the timsTOF at 5 µm spatial resolution in positive ion mode and 250 laser shots per pixel. ……………………………………………………………………………………………....4

Figure S5. Mass spectra of randomly selected individual pixels spatially correlated with the cytoplasm within the cell (a, b, and c) and the cell membrane (d, e, and f), obtained using the timsTOF instrument at a spatial resolution of 5 µm in positive ion mode. The blue asterisk represents actin (*m/z* 1198.71), while the red asterisk represents hnRNP A1 protein (*m/z* 1628.78). …..……………………………………………………………..…………………5

Table S1. List of MALDI MSI peptide-like features identified via LC-MS/MS with corresponding peptide sequence, protein ID, and protein name. Green indicates the presence of the peptide peak in the LC-MS/MS dataset, while red indicates its absence.

m/z	Present in LC/MSMS	Peptide Sequence	Protein ID	Protein name
842.51	red	[R]VATVSLPR[S]	P00761	Trypsin (Porcine)
870.54	red	[R]VATVSLPR[S] - N,N-dimethyl derivative	P00761	Trypsin (Porcine)
976.45	green	[K]AGFAGDDAPR[A]	P60709	Actin
1013.44	green	[R]GGNFGFGDSR[G]	P22626	Heterogeneous nuclear ribonucleoproteins A2/B1
1025.61	green	[K]IGGIGTVPVGR[V]	P68104	Elongation factor 1-alpha 1
1045.56	red	[K]LSSPATLNSR[V]	P00761	Trypsin (Porcine)
1052.51	green	[K]NTPAFFAER[L]	P50995	Annexin A11
1120.52	green	[R]GMGPGTPAGYGR[G]	P23246	Splicing factor, proline- and glutamine-rich
1171.58	green	[R]HQGVMVGMGQK[D]	P60709	Actin
1187.64	green	[K]LRDLEDSLAR[E]	P02545	Prelamin-A/C
1198.71	green	[R]AVFPSIVGRPR[QH]	P60709	Actin
1236.66	green	[R]YLAEVATGEKR[A]	P61981	14-3-3 protein gamma
1248.62	green	[R]FACHSASLTVR[N]	Q15233	Non-POU domain-containing octamer-binding protein
1325.75	green	[R]DNIQGITKPAIR[R]	P62805	Histone H4
1341.67	green	[R]FGQGGAGPVGGQGPR[G]	P23246	Splicing factor, proline- and glutamine-rich
1377.63	green	[R]GGGGNFGPGPGSNFR[G]	P22626	Heterogeneous nuclear ribonucleoproteins A2/B1
1383.61	green	[KR]GASQAGMTGYGMPR[Q]	P37802	Transgelin-2
1377.63	green	[R]GGGGNFGPGPGSNFR[G]	P22626	Heterogeneous nuclear ribonucleoproteins A2/B1
1428.71	green	[R]SLYASSPGGVYATR[S]	P08670	Vimentin
1453.78	green	[K]SGEVLVNVKEHSR[Q]	Q13347	Eukaryotic translation initiation factor 3 subunit I
1495.79	green	[R]TYSLGSALRPSTSR[S]	P08670	Vimentin
1628.78	green	[R]SSGPYGGGGQYFAKPR[N]	P09651	Heterogeneous nuclear ribonucleoprotein A1
1649.84	green	[R]CGPLIDLCRGPHVR[H] - 2 X N,N-dymethyl derivative	P26639	Threonine-tRNA ligase 1
1694.70	green	[R]NQGGYGGSSSSSSYGSGR[R]	P09651	Heterogeneous nuclear ribonucleoprotein A1

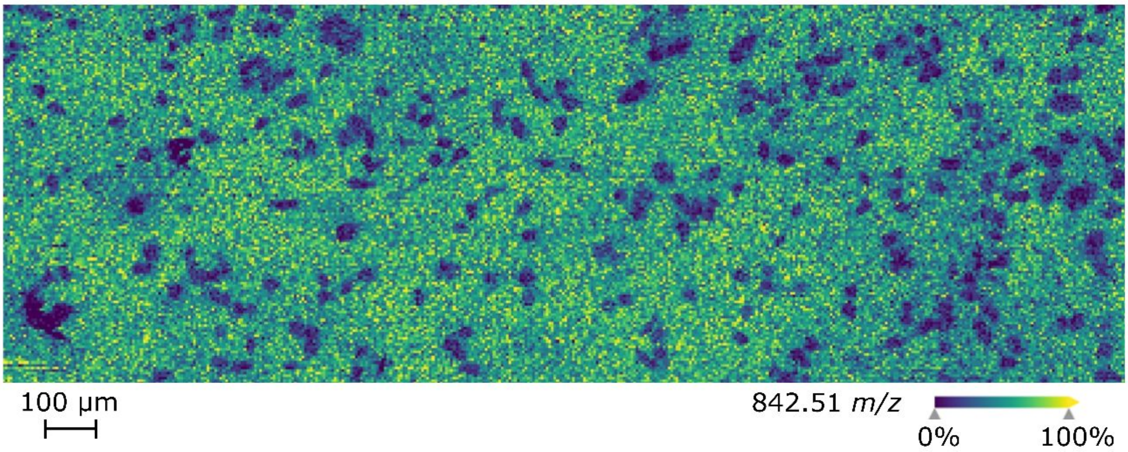

Figure S1. Spatial mapping of the trypsin autolysis peak at *m/z* 842.51. Uniform slide coverage of trypsin was achieved with the HTX M3+ sprayer. The regions devoid of signal on the MALDI image correspond to individual cells cultivated on an ITO slide, where the autolysis rate is comparatively slower due to the presence of other proteins. The timsTOF flex instrument was used to acquire this MALDI MSI data at a pixel size of 5 µm in positive mode.

The CHCA matrix was sprayed with an HTX M3+ sprayer (HTX Technologies, Carrboro, NC, USA). Spraying parameters were as follows: temperature = 75 ˚C, nozzle velocity = 1200 mm/min, flow rate = 120 µL/min, CHCA concentration = 10 mg/mL, solvent = 70% ACN + 1%TFA, number of passes = 4, track spacing = 1.5 mm, nitrogen gas pressure of 10 psi.

In Figure S2, we observed significant delocalization using the optimized matrix spraying protocol, which made it challenging to measure single cells at a 5 µm spatial resolution. The delocalization caused cells to look merged on images, making it hard to differentiate between them. Additionally, compared to the sublimation protocol, we could not observe any spatial distributions within the cells themselves. Furthermore, due to the delocalization, cells appeared larger and more distorted than they are, making it nearly impossible to make any meaningful

connections with cell morphology. Therefore, we developed a sublimation-based matrix application protocol to enable high-spatial resolution MALDI MSI measurements.

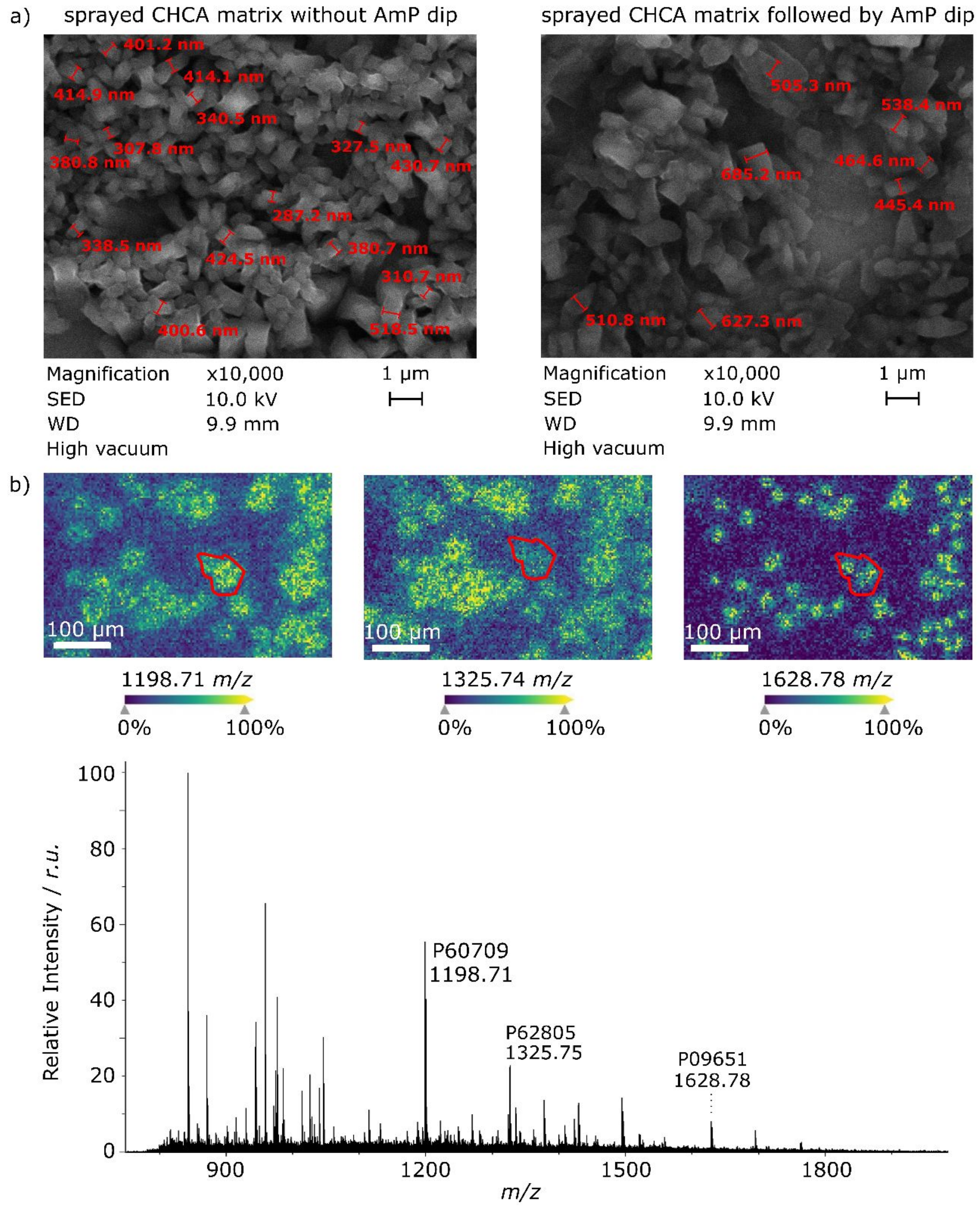

Figure S2. (a) Images of the CHCA crystals after spraying with and without the AmP dip. (b) MALDI mass spectrometry images of single MDA-MB-231 breast cancer cells acquired with the timsTOF at 5 µm spatial resolution in positive ion mode and 250 laser shots per pixel. (c) The average mass spectrum that corresponds to the selected ROI based on 240 averaged mass spectra.

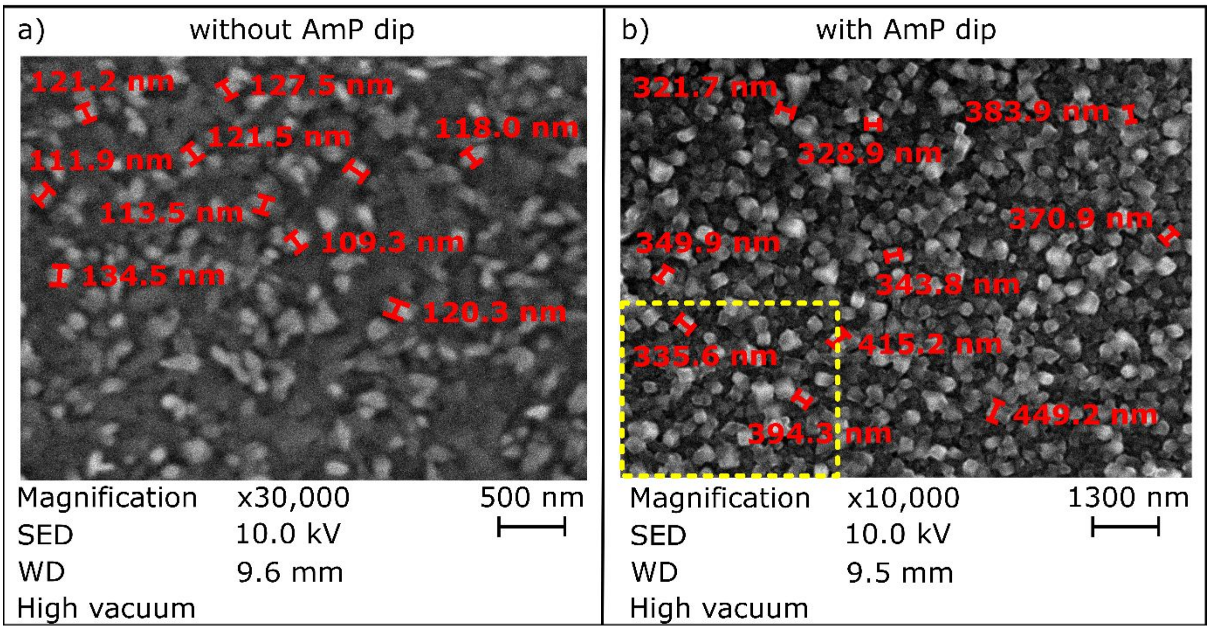

Figure S3. (a) Images of the CHCA crystals after sublimation and no AmP dip. The image corresponds to Figure 2a, presenting measurements of matrix crystals. (b) Images of the CHCA matrix crystals after sublimation and AmP dip. The yellow dashed rectangle corresponds to Figure 2b, presenting measurements of matrix crystals.

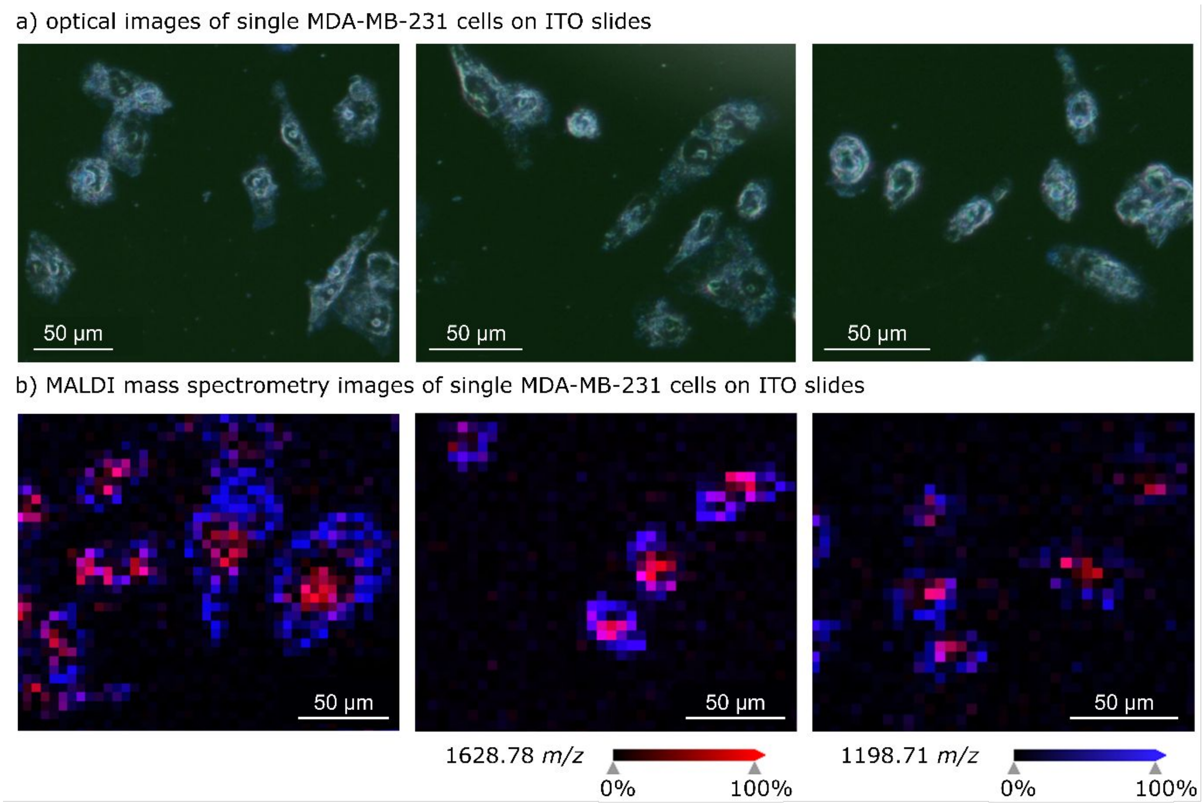

Figure S4. (a) Optical microscopy images of various single MDA-MB-231 cells on ITO-coated slides. Optical images do not depict the same cells as those in MALDI MSI data. (b) MALDI

mass spectrometry images of various single MDA-MB-231 breast cancer cells acquired with the timsTOF at 5 µm spatial resolution in positive ion mode and 250 laser shots per pixel.

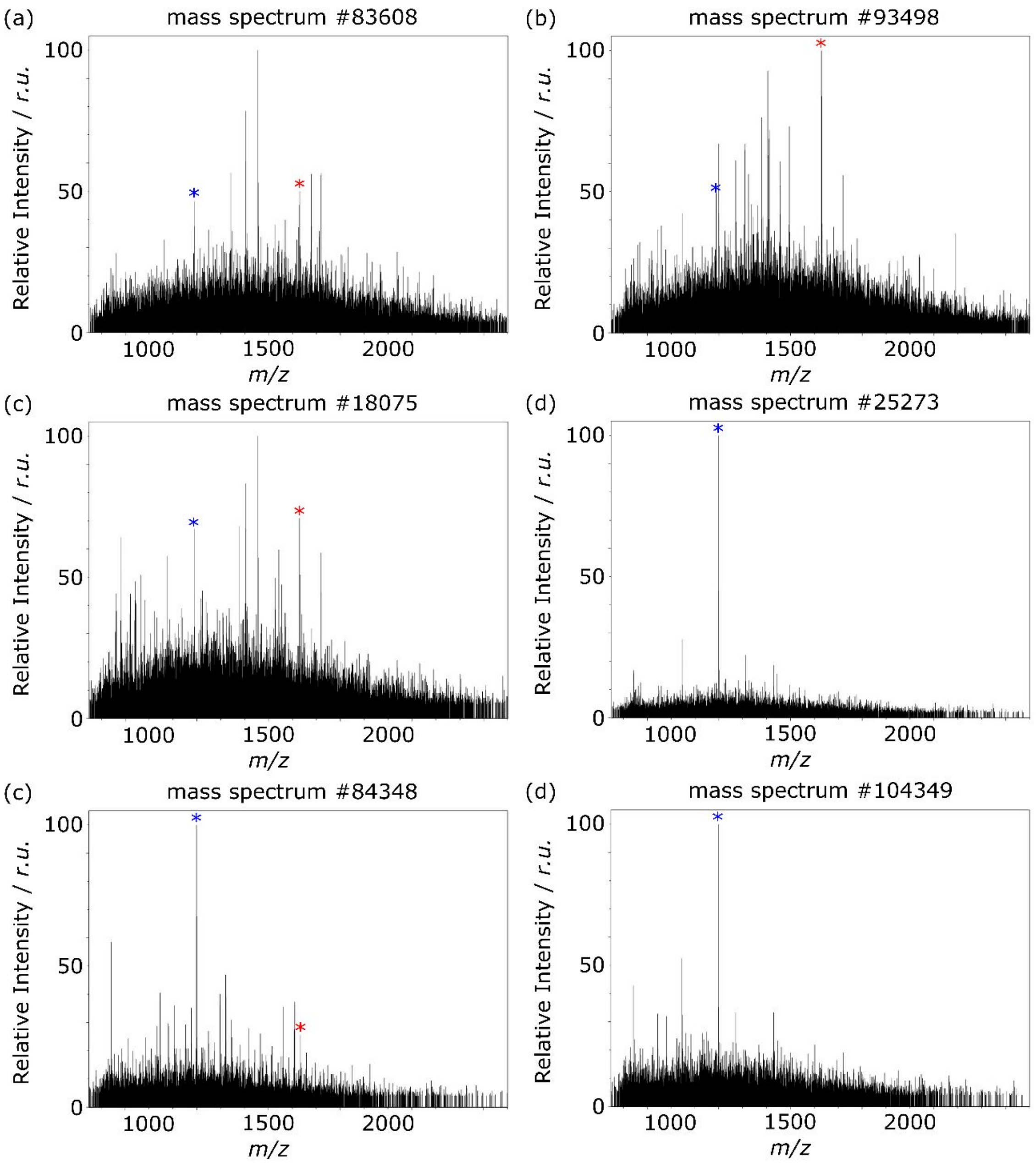

Figure S5. Mass spectra of randomly selected individual pixels spatially correlated with the cytoplasm within the cell (a, b, and c) and the cell membrane (d, e, and f), obtained using the timsTOF instrument at a spatial resolution of 5 µm in positive ion mode. The blue asterisk represents actin (*m/z* 1198.71), while the red asterisk represents hnRNP A1 protein (*m/z* 1628.78).
